# From Hiroshima to Fukushima

**Published:** 2011-08-01

**Authors:** K B Lankarani

**Affiliations:** 1 Health Policy Research Center, Shiraz University of Medical Sciences, Shiraz, Iran

**Keywords:** Bombs, Nuclear power, Earthquake

Japan has been the victim of the worst two nuclear disasters in the world. In the first event, on Monday, August 6th 1945 (just 66 years ago), Hiroshima was bombarded by an American B-29 plane dropping the first atomic bomb on a civilian target [1].The damage was incredible. Recent estimates of death toll in Hiroshima bombing indicates between 90,000 up to 166,000 deaths most of them occurred in the first day of bombing of this city with a resident civilian population of 340,000 [2]. Roughly near 70 % of the city's buildings were completely destroyed and another 7 % were severely damaged [1]. After 3 days another bomb was dropped on Nagasaki with resultant estimated death of 60,000 up to 80,000 out of a population of 250,000 [2]. In both cities most of the deaths were among civilians. It might be interesting to know the bomb in Hiroshima felt directly on a surgical clinic named Shima [1]. Increased risk of cancer was reported within few months among the survivors with the highest risk of leukemia [3][4]. Among those exposed in utero during bombing additional increased risk of congenital anomalies and mental disabilities were noticed [5]. Recently, researchers have shown that risk of many cancers including leukemia as well as non cancer diseases such as cardiovascular diseases were increased in the survivors even several decades after the exposure [3][4]. Going north east from Hiroshima, through only one hour flight there is the beautiful city of: Fukushima. This city became more famous worldwide not for its beauties but for the nuclear disaster that happened this year. On March 11th, 2011 following a 9.0 magnitude earthquake and Tsunami, the Fukushima Daiichi nuclear power complex was damaged. At the time of earthquake one of its six reactors was defueled, two others in the cold shutdown for planned maintenance, but three others were running [6]. Forty seven minutes after the earthquake, ocean's waves with height of 13.1 m reached the plant despite the sea wall which was designed to withstand waves up to 5.7 m. Within minutes the complex was flooded and all the electronically powered safety measures became affected including the cooling system. As a result of natural decay and fission in the absence of any cooling system, overheating of the reactors started with resultant melting of the reactors, multiple hydrogen explosions, fires and water leakage. Field measurements revealed that the levels of iodine-131 and caesium-137 increased not only in the nearby regions but also in the other countries and even other continents. There was a 6 days delay before the radiation reached Tokyo. The radioactive contaminations were reported in milk, vegetable and other food materials. For a period drinking tap water in Tokyo was announced contaminated and even dangerous especially for infants. Trace amounts of radiation, including iodine-131 and caesium-134/137 were reported from both east and west coasts of American continent and not surprisingly from Australia. Although no contamination of food was reported outside of Japan since April 2011, but there are major concerns on the future effects of radioactive debris which have deployed in the Pacific Ocean. Initial assessment of the Japanese authorities on the scale of the disaster was level 4 out of 7 on the International Nuclear Event Scale (INES). This was criticized by many as a misjudgment as one month later the level was raised to 7, the highest on the scale indicating serious hazards for health and environment with wide distribution of the radioactive material. The only prior level 7 event was Chernobyl accident on 1986. Although the Chernobyl disaster might have been larger in the magnitude but the Fukushima event was more complex as multiple reactors were involved.[1][6][7]

Hiroshima and Fukushima are drastic examples of how human lives could be affected by inappropriate use of technology. These sad events need to be analyzed and used as guides for future. In Hiroshima, the lives of thousands of people were lost with a single decision of dropping atomic bombs. The purpose was stopping the world war which was already stopped in Europe without atomic bombing. Was this the only way to stop the war? Did they realize the cost of their decision? Still on the 66th anniversary of the event, the researchers in Japan announced that they do not know the exact impact of the bombing on human lives and environment.[2][3][4] In the second event, the most criticized point was whether there was enough preparedness for such a disaster or could this disaster be predicted in past? However, this should not be translated to no-use of nuclear energy. Instead in facing the climate changes that are affecting the whole world, we must seek more use of nuclear energy but with more global cooperation and vigorous safety standards.[6][7][8]
